# Defining Catastrophic Costs and Comparing Their Importance for Adverse Tuberculosis Outcome with Multi-Drug Resistance: A Prospective Cohort Study, Peru

**DOI:** 10.1371/journal.pmed.1001675

**Published:** 2014-07-15

**Authors:** Tom Wingfield, Delia Boccia, Marco Tovar, Arquímedes Gavino, Karine Zevallos, Rosario Montoya, Knut Lönnroth, Carlton A. Evans

**Affiliations:** 1 Innovación Por la Salud Y Desarrollo (IPSYD), Asociación Benéfica PRISMA, Lima, Perú; 2 Innovation For Health And Development (IFHAD), London, United Kingdom; 3 Infectious Diseases & Immunity, Imperial College London, and Wellcome Trust Imperial College Centre for Global Health Research, London, United Kingdom; 4 The Monsall Infectious Diseases Unit, North Manchester General Hospital, Manchester, United Kingdom; 5 Department of Infectious Disease Epidemiology, London School of Hygiene & Tropical Medicine, London, United Kingdom; 6 Laboratorio de Investigación y Desarrollo, Universidad Peruana Cayetano Heredia, Lima, Perú; 7 Policy Strategy and Innovations, Stop TB Department, World Health Organization, Geneva, Switzerland; Perelman School of Medicine at the University of Pennsylvania, United States of America

## Abstract

**Background:**

Even when tuberculosis (TB) treatment is free, hidden costs incurred by patients and their households (TB-affected households) may worsen poverty and health. Extreme TB-associated costs have been termed “catastrophic” but are poorly defined. We studied TB-affected households' hidden costs and their association with adverse TB outcome to create a clinically relevant definition of catastrophic costs.

**Methods and Findings:**

From 26 October 2002 to 30 November 2009, TB patients (*n* = 876, 11% with multi-drug-resistant [MDR] TB) and healthy controls (*n* = 487) were recruited to a prospective cohort study in shantytowns in Lima, Peru. Patients were interviewed prior to and every 2–4 wk throughout treatment, recording direct (household expenses) and indirect (lost income) TB-related costs. Costs were expressed as a proportion of the household's annual income. In poorer households, costs were lower but constituted a higher proportion of the household's annual income: 27% (95% CI = 20%–43%) in the least-poor houses versus 48% (95% CI = 36%–50%) in the poorest. Adverse TB outcome was defined as death, treatment abandonment or treatment failure during therapy, or recurrence within 2 y. 23% (166/725) of patients with a defined treatment outcome had an adverse outcome. Total costs ≥20% of household annual income was defined as catastrophic because this threshold was most strongly associated with adverse TB outcome. Catastrophic costs were incurred by 345 households (39%). Having MDR TB was associated with a higher likelihood of incurring catastrophic costs (54% [95% CI = 43%–61%] versus 38% [95% CI = 34%–41%], *p*<0.003). Adverse outcome was independently associated with MDR TB (odds ratio [OR] = 8.4 [95% CI = 4.7–15], *p*<0.001), previous TB (OR = 2.1 [95% CI = 1.3–3.5], *p* = 0.005), days too unwell to work pre-treatment (OR = 1.01 [95% CI = 1.00–1.01], *p* = 0.02), and catastrophic costs (OR = 1.7 [95% CI = 1.1–2.6], *p* = 0.01). The adjusted population attributable fraction of adverse outcomes explained by catastrophic costs was 18% (95% CI = 6.9%–28%), similar to that of MDR TB (20% [95% CI = 14%–25%]). Sensitivity analyses demonstrated that existing catastrophic costs thresholds (≥10% or ≥15% of household annual income) were not associated with adverse outcome in our setting. Study limitations included not measuring certain “dis-saving” variables (including selling household items) and gathering only 6 mo of costs-specific follow-up data for MDR TB patients.

**Conclusions:**

Despite free TB care, having TB disease was expensive for impoverished TB patients in Peru. Incurring higher relative costs was associated with adverse TB outcome. The population attributable fraction indicated that catastrophic costs and MDR TB were associated with similar proportions of adverse outcomes. Thus TB is a socioeconomic as well as infectious problem, and TB control interventions should address both the economic and clinical aspects of this disease.

*Please see later in the article for the Editors' Summary*

## Abstract 2

Tom Wingfield and colleagues investigate the relationship between catastrophic costs and tuberculosis outcomes for patients receiving free tuberculosis care in Peru.

*Please see later in the article for the Editors' Summary*

## Editors' Summary

**Background:**

Caused by the infectious microbe *Mycobacterium tuberculosis*, tuberculosis (or TB) is a global health problem. In 2012, an estimated 8.6 million people fell ill with TB, and 1.3 million were estimated to have died because of the disease. Poverty is widely recognized as an important risk factor for TB, and developing nations shoulder a disproportionate burden of both poverty and TB disease. For example, in Lima (the capital of Peru), the incidence of TB follows the poverty map, sparing residents living in rich areas of the city while spreading among poorer residents that live in overcrowded households.

The Peruvian government, non-profit organizations, and the World Health Organization (WHO) have extended healthcare programs to provide free diagnosis and treatment for TB and drug-resistant strains of TB in Peru, but rates of new TB cases remain high. For example, in Ventanilla (an area of 16 shantytowns located in northern Lima), the rate of infection was higher during the study period, at 162 new cases per 100,000 people per year, than the national average. About one-third of the 277,895 residents of Ventanilla live on under US$1 per day.

**Why Was This Study Done?:**

Poverty increases the risks associated with contracting TB infection, but the disease also affects the most economically productive age group, and the income of TB-affected households often decreases post-diagnosis, exacerbating poverty. A recent WHO consultation report proposed a target of eradicating catastrophic costs for TB-affected families by 2035, but hidden TB-related costs remain understudied, and there is no international consensus defining catastrophic costs incurred by patients and households affected by TB. Lost income and the cost of transport are among hidden costs associated with free treatment programs; these costs and their potential impact on patients and their households are not well defined. Here the researchers sought to clarify and characterize TB-related costs and explore whether there is a relationship between the hidden costs associated with free TB treatment programs and the likelihood of completing treatment and becoming cured of TB.

**What Did the Researchers Do and Find?:**

Over a seven-year period (2002–2009), the researchers recruited 876 study participants with TB diagnosed at health posts located in Ventanilla. To provide a comparative control group, a sample of 487 healthy individuals was also recruited to participate. Participants were interviewed prior to treatment, and households' TB-related direct expenses and indirect expenses (lost income attributed to TB) were recorded every 2–4 wk. Data were collected during scheduled household visits.

TB patients were poorer than controls, and analysis of the data showed that accessing free TB care was expensive for TB patients, especially those with multi-drug-resistant (MDR) TB. Total expenses were similar pre-treatment compared to during treatment for TB patients, despite receiving free care (1.1 versus 1.2 times the same household's monthly income). Even though direct expenses (for example, costs of medical examinations and medicines other than anti-TB therapy) were lower in the poorest households, their total expenses (direct and indirect) made up a greater proportion of their household annual income: 48% for the poorest households compared to 27% in the least-poor households.

The researchers defined costs that were equal to or above one-fifth (20%) of household annual income as catastrophic because this threshold marked the greatest association with adverse treatment outcomes such as death, abandoning treatment, failing to respond to treatment, or TB recurrence. By calculating the population attributable fraction—the proportional reduction in population adverse treatment outcomes that could occur if a risk factor was reduced to zero—the authors estimate that adverse TB outcomes explained by catastrophic costs and MDR TB were similar: 18% for catastrophic costs and 20% for MDR TB.

**What Do These Findings Mean?:**

The findings of this study indicate a potential role for social protection as a means to improve TB disease control and health, as well as defining a novel, evidence-based threshold for catastrophic costs for TB-affected households of 20% or more of annual income. Addressing the economic impact of diagnosis and treatment in impoverished communities may increase the odds of curing TB.

Study limitations included only six months of follow-up data being gathered on costs for each participant and not recording “dissavings,” such as selling of household items in response to financial shock. Because the study was observational, the authors aren't able to determine the direction of the association between catastrophic costs and TB outcome. Even so, the study indicates that TB is a socioeconomic as well as infectious problem, and that TB control interventions should address both the economic and clinical aspects of the disease.

**Additional Information:**

Please access these websites via the online version of this summary at http://dx.doi.org/10.1371/journal.pmed.1001675.

- The World Health Organization provides information on all aspects of tuberculosis, including the Global Tuberculosis Report 2013
- The US Centers for Disease Control and Prevention has information about tuberculosis
- Médecins Sans Frontières's TB&ME blog provides patients' stories of living with MDR TB
- TB Alert, a UK-based charity that promotes TB awareness worldwide, has information on TB in several European, African, and Asian languages
- More information is available about the Innovation For Health and Development (IFHAD) charity and its research team's work in Peru

## Introduction

Tuberculosis (TB) disease kills 1.4 million per year and remains a major global health problem [1]. Many low- and middle-income countries are unlikely to meet the Millennium Development Goals for reduction of TB disease prevalence and mortality [1]. This is due in part to poorer people experiencing inequitable healthcare provision and access [2] and suffering a disproportionate burden of morbidity and mortality from TB disease [3],[4].

Poverty increases TB risk [5], and TB exacerbates poverty, affecting the most economically productive age group [6]–[8]. Whilst many countries aim to offer “free” TB treatment to their patients, this free treatment may cover only some diagnostic tests and anti-mycobacterial medications. Patients and their households may incur hidden costs, be they direct “out of pocket” expenses such as for transport, symptom-relieving medicines, or additional food, or indirect expenses associated with lost income [8]–[12].

In its post-2015 Global Strategy and Targets for Tuberculosis Prevention, Care, and Control at the 67th World Health Assembly in May 2014, the World Health Organization adopted a target of eradicating catastrophic costs for TB-affected families by 2035 [13]. However, hidden TB-related costs remain understudied, and consensus about defining catastrophic costs is awaited [5],[13]–[16]. Some catastrophic costs definitions have incorporated symptoms of financial shock and coping mechanisms [17],[18]. Others have used operational thresholds of total costs of 10%–25% of a household's annual income [16],[19],[20] or 40% or more of a household's “capacity to pay” [21],[22]. Recently, concerns have been raised that the current approach of measuring catastrophic costs using out-of-pocket payments is too narrow because it overlooks lost income and consequently risks misinforming policy-makers [23],[24]. Thus, there is an urgent need to improve indicators of financial risk to better inform health policy guidance [23]–[25]. However, although there is broad agreement that some vulnerable TB-affected households will require social protection (such as socioeconomic support) to avoid catastrophic costs, more evidence is needed to define such costs and characterise their importance [9],[14],[21]–[28].

We prospectively quantified changes in income and hidden costs prior to and throughout treatment of patients with multi-drug-resistant (MDR) and non-MDR TB in impoverished shantytowns surrounding Lima, Peru. The aims of the study were to better characterise TB-related costs and their association with adverse TB outcome, and to contribute to an evidence-based definition of catastrophic costs that is both clinically and financially relevant. The study hypothesis was that catastrophic costs of TB-affected households are independently associated with adverse TB outcome in TB patients.

## Methods

### Ethical Approval

The internationally accredited ethical committee of the Universidad Peruana Cayetano Heredia approved the project. All interviewed participants gave written informed consent.

### Study Design and Participants

We conducted a prospective cohort study of TB patients and a baseline case-control study comparing them with healthy controls. From 26 October 2002 to 30 November 2009, in collaboration with the Peruvian National Tuberculosis Control Program, all consecutive patients with laboratory-proven pulmonary TB were invited to participate in the study. All interactions between the research team and the participants occurred during household visits. Until 30 November 2012, patients were followed-up for recurrent TB by monitoring TB treatment records and revisiting each household approximately every 3 y to enquire about TB diagnoses. From 21 December 2006 to 9 December 2007, control households were selected from an up-to-date satellite map using random number tables and were invited to participate during a household visit. In the case that all potential control participants in the household were unavailable or declined then the nearest neighbouring household was instead invited to participate. Controls were not matched to cases because the study aimed to characterise the effect of relevant exposures including sex, age, and socioeconomic position on the outcome variables of catastrophic costs and adverse TB outcome. The inclusion criterion in both cases and controls was age more than 15 y. Exclusion criteria included declining or being unable to give informed written consent. Both for the cohort and the baseline case-control study, the sample size was opportunistic, and consequently no power calculations were performed.

### Study Setting

The study was conducted in Ventanilla, 16 peri-urban contiguous shantytowns in north Lima, Peru, with an estimated population of 277,895 people and frequent poverty (32% of inhabitants live on ≤US$1 per day). During the study period, the annual TB notification rate in Ventanilla was 162 new cases per 100,000 people per year, higher than the rest of the country, at 106 per 100,000 people annually [29].

TB was treated by the National Tuberculosis Control Program in community health posts where sputum smear was offered free of charge to all patients, and chest radiographs to selected patients. TB patients received their anti-TB directly observed therapy (DOT) free of charge at their local health post, administered by the national TB program.

### Variables

Operational definitions of the key study variables (TB disease, TB treatment phases, TB adverse outcome, and TB costs) are summarised in Box 1.

**Box 1. Glossary of Operational Definitions of TB Disease, Treatment, Outcome, and Costs**

**TB Disease**

*MDR TB:* patients initially prescribed an MDR treatment regimen or who had a sputum test positive for MDR TB by the microscopic-observation drug-susceptibility (MODS) assay or the proportions assay

*Non-MDR TB:* all patients recruited to the study not meeting the definition for MDR TB

TB Treatment Phases*

*Pre-treatment:* the period of time from self-reported onset of TB-related symptoms until treatment initiation

*Intensive treatment phase:* the first two consecutive months of TB treatment

*Continuation treatment phase:* the four consecutive months immediately following the intensive treatment phase

*During treatment:* the period of time spanning from the beginning of the intensive treatment phase to the end of continuation treatment phases

*Entire illness:* the period of time from the onset of TB-related symptoms to the end of the continuation treatment phase

**TB Treatment Outcome**

*Adverse TB outcome:* patients who died during treatment (irrespective of cause), abandoned treatment, had treatment failure, or had recurrent TB disease within 30 mo of starting TB treatment

*Good TB outcome:* patients who were declared cured by the TB program and had no recurrence of TB disease within 30 mo of starting treatment

*Undefined TB outcome:* patients who were transferred by the national TB program to another health post outside of the study site or were lost to follow-up

**TB Costs**

*Direct (“out of pocket”) expenses:* the sum of the direct medical expenses and direct non-medical expenses

*Direct medical expenses:* costs of medical examinations and medicines

*Direct non-medical expenses:* costs of natural remedies, TB-care-related transport, extra food, and other miscellaneous expenses

*Lost income (indirect expenses):* the income the patient estimated that the household lost due to TB illness or TB-related time off work (such as attending clinics) (a) from symptom onset until the recruitment interview and (b) from the previous interview date until subsequent interviews

*Total expenses:* direct expenses plus lost income

*Earnings:* the monthly money actually received by the household

*Income:* the monthly money that would have been earned by the household if it were not TB-affected (earnings *plus* lost income)

*Catastrophic costs threshold:* the threshold at which total household expenses as a proportion of annual income were most strongly associated with adverse TB outcome; the strength of the association was assessed by the highest sensitivity, specificity, and population attributable fraction for adverse outcome

*These treatment definitions apply to all TB patients, irrespective of whether they had MDR or non-MDR TB.

Patients were defined as having MDR TB if they were initially prescribed an MDR TB treatment regimen or sputum testing was positive for MDR TB by the microscopic-observation drug-susceptibility (MODS) assay or the proportions assay. All other patients recruited to the study were defined as having non-MDR TB.

For both patients with MDR and patients with non-MDR TB, stages of treatment were operationally defined as follows: “pre-treatment” was from self-reported onset of TB-related symptoms until treatment initiation; “intensive treatment phase” was the first 2 mo of TB treatment; “continuation treatment phase” was the 4 mo immediately following the “intensive treatment phase”; “during treatment” was the period of time from the start of the “intensive treatment phase” to the end of the “continuation treatment phase”; and “entire illness” was the period of time from TB-related symptom onset to the end of the “continuation treatment phase”.

Early TB treatment outcome for each patient was assessed by the national TB program at the time of treatment cessation and was not influenced by this research. These early TB treatment outcome assessments were based on sputum microscopy results that are insensitive to treatment failure [30]–[32]. Therefore, we also collaborated with the national TB program in continuous surveillance of national TB program treatment records and revisited each patient in their home to check for TB recurrence, which we defined as TB retreatment within 30 mo from the date that treatment started (in most cases 2 y from treatment cessation). We defined good TB outcome as cure without recurrence. We defined adverse TB outcome as death during treatment, treatment abandonment, treatment failure, or recurrence. Patients who were transferred by the national TB program to another health post outside of the study site or were lost to follow-up were considered to have undefined outcome.

### Data Source and Measurement

A questionnaire was developed locally, piloted, refined, and then used to interview patients and collect socio-demographic data concerning household income and expenses throughout TB illness (Questionnaire S1). Interviews were conducted at baseline with both TB patients and controls. For patients, this baseline interview occurred prior to or at the time that treatment commenced. The baseline interview (but not subsequent interviews) included detailed assessment of household assets ownership, access to basic services, and education level. Patients were subsequently interviewed after 2, 4, 6, 8, 12, 16, 20, and 24 wk of treatment. At the baseline and all subsequent interviews, questions characterised earnings, income, expenses, employment (paid or unpaid), number of days unable to work due to illness, additional household food expenditure due to TB illness, and crowding. Household debts were assessed at recruitment and subsequently at 24 wk of treatment.

As in previous research, TB-related costs were categorised as “direct expenses” [6],[9],[30],[33] and “lost income” [6],[33],[34] incurred since the previous interview. All costs and incomes were quantified in cash amounts in Peruvian Soles (PEN) (US$1 on average equivalent to 2.9 PEN during the study period). Inflation and especially exchange rates varied considerably during the study period, so actual costs were reported without adjustments in order to be more informative to users including policy-makers. Table S6 shows annual inflation in Peru and average annual exchange rates for 2002–2009. To further facilitate interpretation internationally, costs were also expressed as the proportion of the average monthly income of all patient households in the cohort (termed “monthly incomes”). Also, to assess impact on the patient households, costs were calculated as the proportion of the same household's annual income.

“Direct (out-of-pocket) expenses” included direct medical expenses (medical examinations and prescribed medicines) and direct non-medical expenses (natural non-prescribed remedies, TB-care-related transport, extra food, and other miscellaneous expenses). “Lost income” (indirect expenses) was the income the patient estimated that the household lost due to TB illness or TB-related time off work (such as attending clinics) since the previous interview, measured in Peruvian Soles. Number of days of work lost due to TB illness could not be used to directly calculate lost income because salaried employment with fixed rates of remuneration was uncommon in this setting. “Total expenses” were direct expenses plus lost income. “Earnings” were defined as the monthly money actually received by the household, and “income” was defined as the monthly money earned by the household *plus* lost income. Household debts at recruitment and total household debts (sum of debts at recruitment plus debts at 24 wk of recruitment) included both formal debts (e.g., bank loans) and informal debts (e.g., money borrowed from friends and family).

For all participants, height and weight were measured and body mass index (BMI) was calculated. Poverty was measured using a composite household poverty index in arbitrary units derived by principal component analysis from 13 variables, as previously described [35].

A threshold for catastrophic costs was calculated by plotting the sensitivity, specificity, and population attributable fraction for adverse TB outcome against total household expenses as a proportion of annual income. In order to assess the strength of this new definition of catastrophic costs and in accordance with relevant recent studies [16],[20], a sensitivity analysis was also performed comparing the association of other existing catastrophic costs thresholds (including total expenses equal to or greater than 10%, 15%, or 25% of annual income) with adverse TB outcome.

### Data Analysis

Continuous data were summarised by their arithmetic means and their 95% confidence intervals (CIs) whether the data were Gaussian or non-Gaussian because this approach is considered to be robust for health economics data analysis [7],[36],[37]. Furthermore, because of the skewed nature of some expenditure data, most median values were zero or close to zero, limiting the descriptive usefulness of presenting median values. Any direct expenses, lost income, or annual income recorded as “zero” or missing was replaced with 0.5 PEN per day (i.e., the midpoint of zero and the lowest unit of measurement, 1 PEN). Means were compared with the Student's *t*-test. Categorical data were summarised as proportions with 95% CIs and were compared with the *z*-test of proportions. Univariable regression analyses examining differences between patients with MDR and non-MDR TB and controls were adjusted for sex because of under-recruitment of male controls due to their availability.

The association between catastrophic costs and adverse TB outcome was explored through both univariable and multivariable analysis to determine odds ratios (ORs). The likelihood ratio test was used to test for trend and interaction between variables. Non-Gaussian continuous variables such as total costs as a proportion of annual income were transformed to their base-10 logarithm for regression analysis. All the variables associated (*p*<0.15) with adverse TB outcome in univariable analysis and all predetermined presumed confounding variables (age, poverty score, previous TB episode, symptom duration, and current MDR TB) were concurrently included in a multivariable model [38].

Population attributable fractions were calculated using the Stata program “aflogit” function, which computes population attributable fraction estimates while adjusting for the reciprocal confounding effect of covariates on the association of interest. The population attributable fraction of an exposure was interpreted as the proportion of adverse TB outcomes that would be averted by eliminating that exposure, both unadjusted and adjusted for known confounding factors. All *p*-values were two-sided, and statistical analyses were performed using the Stata program (version 10, StataCorp).

## Results

### Participants

During the study period, the Peruvian national TB program within the study site of Ventanilla registered 1,014 patients. We located 99% of these registered TB patients, of whom 95% (*n* = 966) met the inclusion criterion. Of these eligible patients, 1% (*n* = 10) declined, and 8% (*n* = 80) were excluded because they completed fewer than half of our planned research interviews; data are presented for the remaining 91% (*n* = 876). 11% (*n* = 93) of patients recruited had MDR TB. 487 controls were also recruited and had only a baseline interview. The characteristics of the study population are summarised in Table 1.

**Table 1:** Study population baseline data.

Category	Characteristic	Controls	TB Patients	*p*-Value*	Non-MDR TB	MDR TB	*p*-Value*
**Total participants**		487	876		783	93	
**Demographics**	**Age (years); mean**	34	31	0.001	31	31	0.8
	[SD]	[30–48]	[18–44]		[30–32]	[17–45]	
	**Male; percent**	37	59	<0.001	59	59	0.9
	[95% CI]	[33–41]	[55–62]		[55–62]	[49–69]	
**Health and finances**	**Completed secondary school; percent**	46	44	0.3	45	36	0.1
	[95% CI]	[41–50]	[41–47]		[42–49]	[26–46]	
	**Household crowding above mean; percent**	66	57	0.07	57	61	0.5
	[95% CI]	[59–72]	[54–61]		[53–60]	[51–71]	
	**People per house; mean**	5.1	4.9	0.8	4.9	4.9	0.9
	[IQR]	[4.6–5.6]	[4.8–5.0]		[4.7–5.0]	[4.5–5.4]	
	**BMI (kg/m^2^); mean**	26	21	<0.001	21	21	0.3
	[95% CI]	[25–26]	[21–22]		[21–22]	[20–21]	
	**Previous TB; percent**	5.4	18	<0.001	15	40	<0.001
	[95% CI]	[3.3–7.4]	[15–20]		[13–18]	[30–50]	
	**Monthly earnings** ** **pre-treatment; mean**	651 (1.40)	510 (1.09)	<0.001	511 (1.09)	497 (1.07)	0.8
	[95% CI]	[595–707]	[481–539]		[482–540]	[381–613]	
	**Monthly earnings** ** **during treatment; mean**		434 (0.93)		436 (0.94)	418 (0.90)	0.6
	[95% CI]		[415–453]		[416–456]	[341–495]	
	**Monthly earnings** ** **in intensive phase; mean**		379 (0.81)		379 (0.81)	376 (0.81)	0.9
	[95% CI]		[358–400]		[357–401]	[295–457]	
	**Monthly earnings** ** **in continuation phase; mean**		454 (0.97)		457 (0.98)	424 (0.91)	0.4
	[95% CI]		[431–477]		[434–480]	[339–509]	
	**Debt** *** **; mean**	812 (1.7)	383 (0.82)	0.004	377 (0.81)	435 (0.93)	0.7
	[95% CI]	[507–1117]	[292–474]		[283–471]	[87–872]	
	**Not in paid work; percent**	63	81	<0.001	80	90	<0.03
	[95% CI]	[56–69]	[79–84]		[77–83]	[84–96]	
	**Poverty score above control mean; percent**	51	58	<0.02	58	60	0.8
	[95% CI]	[47–56]	[55–61]		[54–61]	[50–70]	
**Current TB**	**Symptom duration (days); mean**		55		52	83	<0.001
	[SD]		[0–127]		[0–118]	[0–192]	
	**Too unwell to work (days); mean**		19		18	29	0.004
	[SD]		[0–51]		[0–47]	[0–81]	
	**Time to health centre (minutes); mean**		13		13	13	0.9
	[SD]		[0–30]		[0–30]	[0–33]	
	**MDR TB; percent**		11				
	[95% CI]		[9–13]				

All data are at individual level and pre-treatment except where indicated.

*Univariable regression adjusted for sex. The first *p*-value column corresponds to comparison of controls (*n* = 487) with all TB patients regardless of MDR status (*n* = 876). The second *p*-value column corresponds to comparison of patients with non-MDR TB (*n* = 783) versus MDR TB (*n* = 93).

**Household earnings per month during different treatment stages represented as mean Peruvian Soles and, in parentheses, as a proportion of TB patients' mean monthly household earnings throughout entire illness. Confidence intervals are those of mean monthly earnings in Peruvian Soles.

***Debt at recruitment represented as mean Peruvian Soles and, in parentheses, as a proportion of TB patients' mean monthly household earnings. Debt at recruitment was used in the final multivariable regression model rather than total debt (sum of debt at recruitment plus debt at 24 wk of treatment) because only 461 patients had 24-wk debt data available.

CI, confidence interval; IQR, interquartile range; SD, standard deviation.

### Descriptive Data

TB patients were more likely than controls to be younger (mean age 31 [95% CI = 18–44] versus 34 [95% CI = 30–48] y old, *p* = 0.001), to be male (59% [95% CI = 5%5–62%] versus 37% [95% CI = 33%–41%] male, *p*<0.001), to have a lower BMI (21 [95% CI = 21–22] versus 26 [95% CI = 25–26] kg/m^2^, *p*<0.001), to have lower earnings (510 [95% CI = 481–539] versus 651 [95% CI = 595–707] PEN, *p*<0.001), to not be in paid work at recruitment (81% [95% CI = 79%–84%] versus 63% [95% CI = 56%–69%], *p*<0.001), and to have had a previous TB episode (18% [95% CI = 15%–20%] versus 5.4% [95% CI = 3.3%–7.4%] of individuals, *p*<0.001). Patients with MDR TB were more likely than patients with non-MDR TB to have had a previous TB episode (40% [95% CI = 30%–50%] versus 15% [95% CI = 13%–18%] of individuals, *p*<0.001), to have longer pre-treatment symptom duration (83 [95% CI = 0–192] versus 52 [95% CI = 0–118] d, *p*<0.001), to not be in paid work (90% [95% CI = 84%–96%] versus 80% [95% CI = 77%–83%] of individuals, *p*<0.03), and to have had more days not working pre-treatment due to TB-related illness (29 [95% CI = 0–81] versus 18 [95% CI = 0–47] d, *p* = 0.004).

Patients earned more per month pre-treatment than during treatment (510 [95% CI = 481–539] versus 434 [95% CI = 415–453] PEN, *p*<0.001; Table 2) or the continuation treatment phase (454 [95% CI = 431–477] PEN, *p*<0.001), and earned least during the intensive treatment phase (379 [95% CI = 358–400] PEN, *p*<0.001). During all treatment phases, patients with MDR TB tended to earn less than patients with non-MDR TB (Table 2). Household debts at recruitment were greater in controls than in patients with TB (812 [95% CI = 507–1117] versus 383 [95% CI = 292–474] PEN, *p* = 0.004), but there was no difference between the household debts of patients with MDR and non-MDR TB (497 [95% CI = 381–613] versus 511 [95% CI = 482–540] PEN, *p* = 0.8). Household debts decreased from 383 (95% CI = 292–474) PEN at recruitment to 296 (95% CI = 176–414) PEN at 24 wk of treatment. Households with total debt above the cohort median were more likely to incur catastrophic costs (OR 1.58 [95% CI = 1.17–2.14], *p* = 0.003). Households with above-cohort-median increase in debt from recruitment to 24 wk were also more likely to incur catastrophic costs (OR 1.74 [95% CI = 1.10–2.77], *p* = 0.003).

**Table 2:** Comparison of mean monthly earnings of patient households by treatment stage.

Treatment Stage	Earnings as Mean Monthly Peruvian Soles (Proportion of Mean Monthly Average Cohort Earnings) [95% CI]					
	All Patients (*n* = 876)	*p*-Value	Non-MDR Patients (*n* = 783)	*p*-Value	MDR Patients (*n* = 93)	*p*-Value
Pre-treatment (*n* = 876)	510 (1.09)		511 (1.09)		497 (1.07)	
	[481–539]		[482–540]		[381–613]	
During treatment (*n* = 876)	434 (0.93)	<0.001	436 (0.94)	<0.001	418 (0.90)	0.09
	[415–453]		[416–456]		[341–495]	
Intensive treatment phase (*n* = 876)	379 (0.81)	<0.001	379 (0.81)	<0.001	376 (0.81)	0.1
	[358–400]		[357–401]		[295–457]	
Continuation treatment phase (*n* = 876)	454 (0.97)	0.001	457 (0.98)	0.0003	424 (0.91)	0.1
	[431–477]		[434–480]		[339–509]	

Mean monthly earnings are shown in Peruvian Soles and in parentheses as a proportion of mean monthly average cohort earnings. Confidence intervals in brackets below earnings are those of mean monthly earnings in Peruvian Soles. *p*-Values represent the difference in earnings between treatment stages by Student's *t*-test. From left to right, data and *p*-values correspond to all patients, patients with non-MDR TB, and patients with MDR TB. In addition to the *p*-values shown in the table, there was also a significant difference between the intensive and continuation treatment phases in all patients (*p*<0.001) and in patients with non-MDR TB (*p*<0.001) but not in patients with MDR TB (*p* = 0.1).

### Outcome Data

725 (83%) patients had a defined TB outcome at follow-up. Of these patients, 166 (23%) had an adverse TB outcome, 40% (*n* = 67) due to treatment abandonment, 22% (*n* = 36) due to treatment failure, 12% (*n* = 15) due to death during treatment, and 26% (*n* = 48) due to TB recurrence.

### Costs Data

#### Direct expenses and lost income

**Figure 1:**
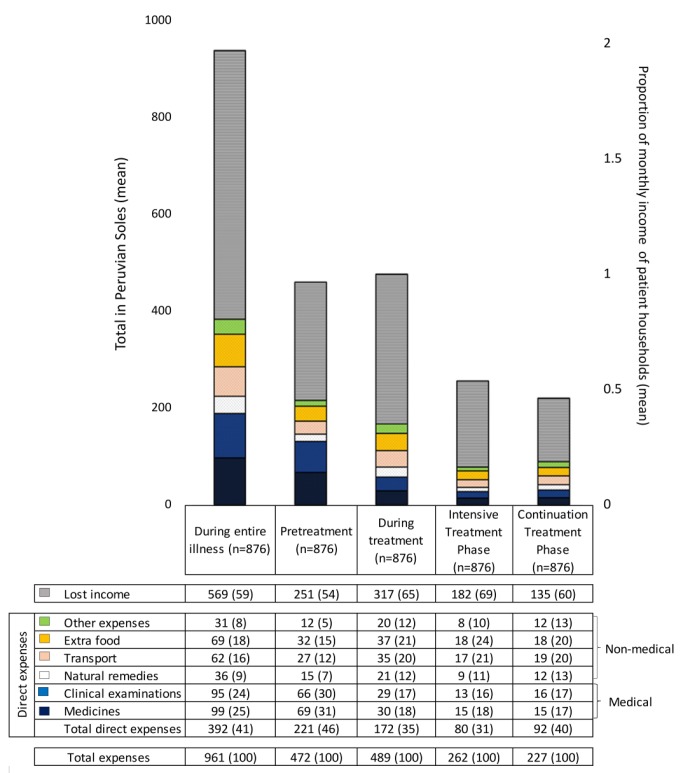
Lost income, direct expenses, and total expenses by treatment stage in mean Peruvian Soles (PEN) and as a proportion of mean monthly household income. The top row of data in the table below the bar graph shows lost income in mean Peruvian Soles and, in parentheses, as a percentage of total expenses. The next six rows show direct expenses in mean Peruvian Soles and, in parentheses, as a percentage of total direct expenses. Medical expenses are defined as the sum of direct expenses for medicines (blue bar) and clinical exams (dark blue bar); non-medical expenses are defined as the sum of direct expenses for natural remedies, TB-care-related transport, extra food, and other TB-related expenses. The lowermost two rows show total direct expenses (i.e., sum of medicines, clinical exams, natural remedies, transport, extra food, and other expenses) and total expenses in mean Peruvian Soles and, in parentheses, as a percentage of total expenses. *p*-Values represent the difference between treatment stages by Student's *t*-test. 23/876 (2.6%) of the TB patient cohort had direct expenses of 0 PEN, and 14/876 (1.6%) had total expenses of 0 PEN, and thus these zero values were replaced with 0.5 PEN per day. A line chart representation of this graph is available in Figure S1.

Direct expenses and lost income are summarised in Figures 1 and S1. Throughout the entire illness, the proportions of direct expenses that were medical and non-medical were similar (49% [95% CI = 43%–55%] versus 51% [95% CI = 47%–54%] of total direct expenses, *p* = 0.7). Medical expenses were greatest pre-treatment: during this period, medical expenses were higher than non-medical expenses and constituted almost two-thirds of overall direct expenses (0.20 [95% CI = 0.18–0.22] versus 0.13 [95% CI = 0.11–0.15] monthly incomes, *p*<0.001). Conversely, during treatment, non-medical expenses were higher than medical expenses and constituted approximately two-thirds of overall direct expenses (0.22 [95% CI = 0.20–0.24] versus 0.14 [95% CI = 0.12–0.16] monthly incomes, *p*<0.001). Direct expenses were higher pre-treatment than during treatment (0.52 [95% CI = 0.46–0.59] versus 0.41 [95% CI = 0.37–0.44] monthly incomes, *p*<0.001), whereas lost household income was lower pre-treatment than during treatment (0.60 [95% CI = 0.50–0.69] versus 0.75 [95% CI = 0.68–0.82] monthly incomes, *p*<0.005). Lost household income was higher than direct expenses throughout all treatment phases, with the greatest difference during the intensive treatment phase (69% lost income [95% CI = 61%–77%]; Figures 1 and S1).

#### Total expenses

In addition to direct expenses and lost income, total expenses are summarised in Figures 1 and S1. Total expenses were similar pre-treatment and during treatment (1.1 [95% CI = 1.0–1.2] versus 1.2 [95% CI = 1.1–1.2] monthly incomes, *p* = 0.6). Total expenses (1.12 [95% CI = 0.99–1.25] versus 0.62 [95% CI = 0.56–0.68] monthly incomes, *p*<0.001), direct expenses (0.52 [95% CI = 0.46–0.59] versus 0.19 [95% CI = 0.16–0.22] monthly incomes, *p*<0.001), and lost income (0.6 [95% CI = 0.50–0.69] versus 0.43 [95% CI = 0.38–0.48] monthly incomes, *p* = 0.001) were significantly higher pre-treatment than during the intensive treatment phase. Total expenses (0.62 [95% CI = 0.56–0.68] versus 0.54 [95% CI = 0.49–0.59] monthly incomes, *p* = 0.01) and lost income (0.43 [95% CI = 0.38–0.48 versus 0.32 [95% CI = 0.28–0.36] monthly incomes, *p*<0.001) were higher in the intensive than the continuation treatment phase, but there was no difference in direct expenses between these treatment phases (0.19 [95% CI = 0.16–0.22] versus 0.22 [95% CI = 0.20–0.23] monthly incomes, *p* = 0.07). When total expenses were examined per month, monthly total expenses for the intensive treatment phase were approximately double those of the continuation treatment phase.

#### Poverty and expenses

**Figure 2:**
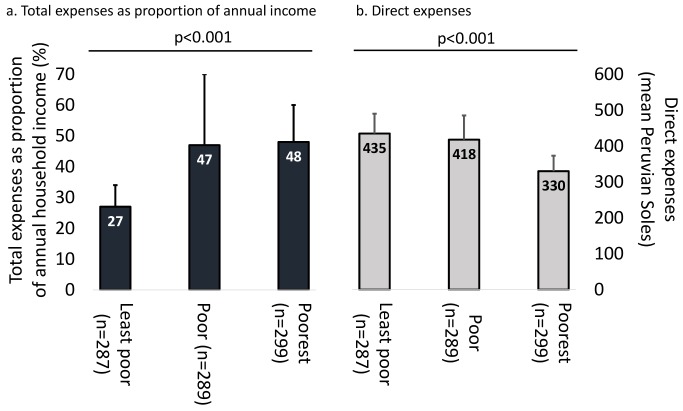
Expenses and economic burden of TB illness across poverty terciles. (A) Total expenses as proportion of annual income. (B) Direct expenses. *p*-Values represent Pearson's coefficient of trend. Bars represent confidence intervals. The numbers in the three bars of (A) refer to the left-hand *y*-axis of total expenses as a proportion of annual household income. The numbers in the three bars of (B) refer to the right-hand *y*-axis of direct expenses in mean Peruvian Soles.

TB patients were poorer than controls (58% [95% CI = 55%–61%] versus 51% [95% CI = 47%–56%] above control mean, *p*<0.02; Table 1). In poorer households, direct expenses were lower (mean direct expenses of poorest households 330 [95% CI = 287–373], poor households 418 [95% CI = 351–485], and least-poor households 435 [95% CI = 380–490] PEN, *p*<0.001; Figure 2), but total expenses made up a greater proportion of the same household's annual income (poorest households 48% [95% CI = 35%–50%], poor households 47% [95% CI = 24%–70%], and least-poor households 27% [95% CI = 20%–34%], *p*<0.001; Figure 2).

### Main Findings

#### Catastrophic costs

**Figure 3:**
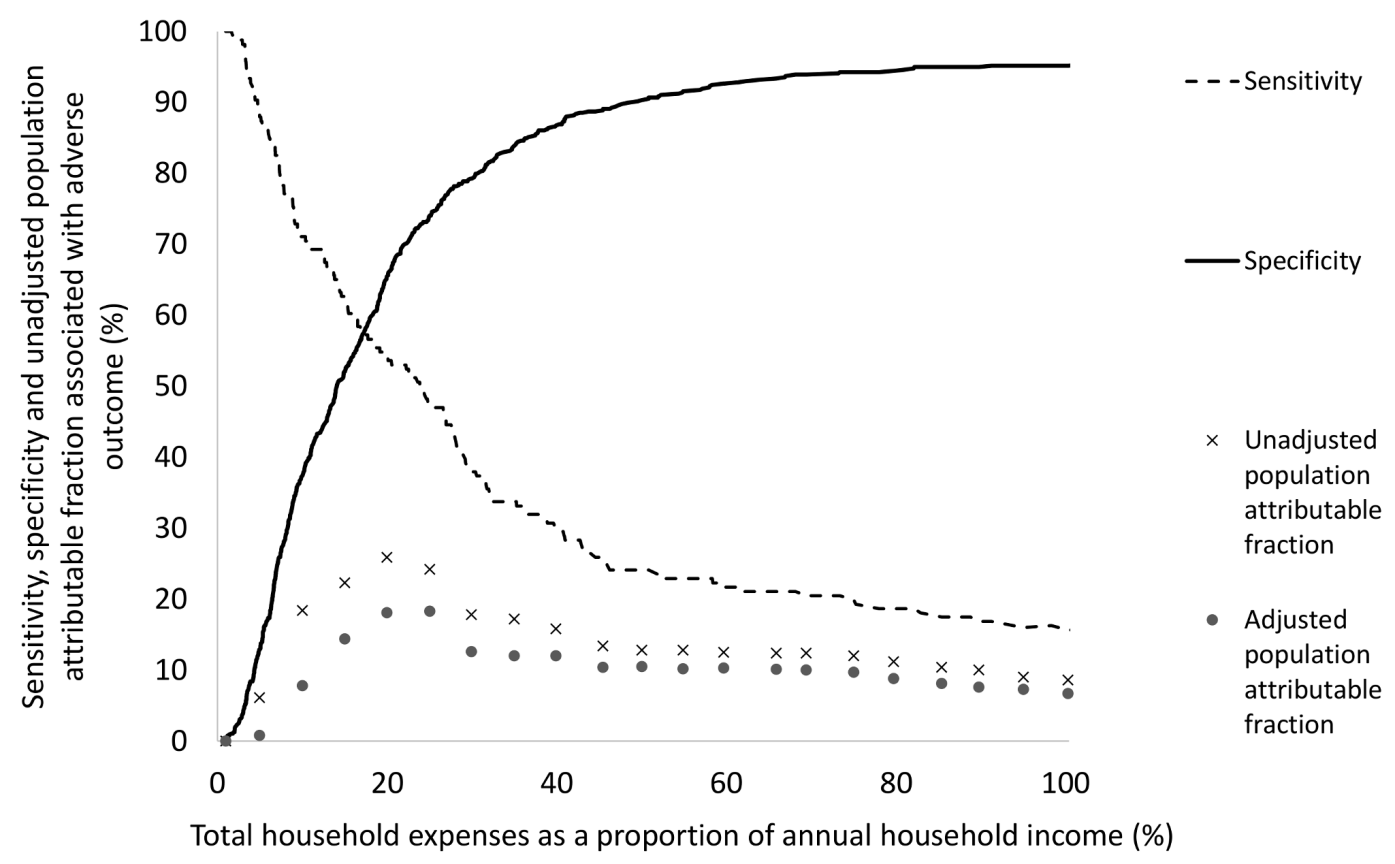
Sensitivity, specificity, and univariable population attributable fraction of the association of total expenses as a proportion of annual income with adverse TB outcome. Total household TB-associated costs were defined as catastrophic when they met or exceeded 20% of household annual income because this threshold had the highest sensitivity, specificity and population attributable fraction for association with adverse outcome.

**Figure 4:**
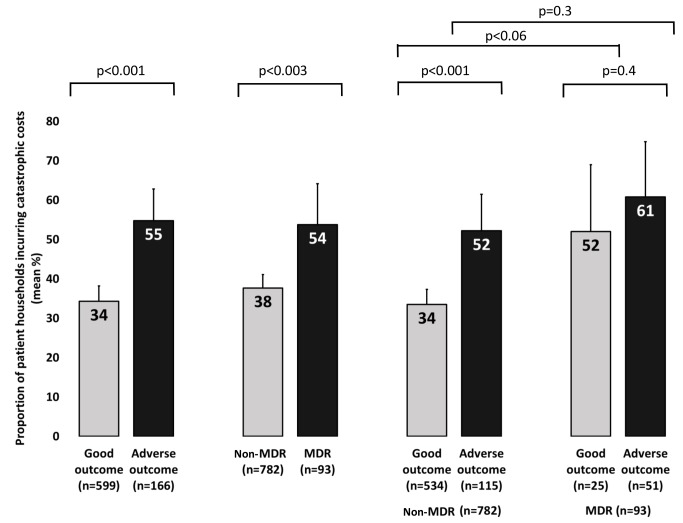
Patient households incurring catastrophic costs by TB resistance profile and adverse TB outcome. Error bars represent 95% confidence intervals. *p*-Values represent association in univariable logistic regression.

A threshold of total expenses ≥20% of annual household income was defined as catastrophic because this threshold had the highest sensitivity, specificity, and population attributable fraction for association with adverse outcome (Figure 3). Catastrophic costs were incurred by 345 households (39%). Incurring catastrophic costs was independently associated with MDR TB (OR 1.61 [95% CI = 0.98–2.64], *p*<0.06), more days not working pre-treatment (OR 1.00 [95% CI = 1.00–1.01], *p* = 0.03), greater debts at recruitment (OR 1.00 [95% CI = 1.00–1.00], *p* = 0.02), being male (OR 2.16 [95% CI = 1.57–2.96], *p*<0.001), being older (OR 1.01 [95% CI = 1.00–1.03], *p* = 0.02), being poorer (OR 1.25 [95% CI = 1.15–1.36], *p*<0.001) and not being in paid employment (OR 1.86 [95% CI = 1.23–2.79], *p* = 0.003; Table 3). Households of patients who had MDR TB were more likely to incur catastrophic costs than households of patients with non-MDR TB (54% [95% CI = 43%–64%] versus 38% [95% CI = 34%–41%], *p*<0.003; Figure 4). When the catastrophic costs multivariable regression analyses were repeated with total costs as a proportion of annual income analysed as a continuous outcome variable (instead of a dichotomous variable: above versus below a threshold indicating catastrophic costs), the results and patterns of significance were similar (Table S1).

**Table 3:** Factors associated with incurring catastrophic costs.

Category	Characteristic	Univariable Logistic Regression		Multivariable Logistic Regression	
		OR	*p*-Value	OR	*p*-Value
**Demographics**	**Age (years)**	1.02	0.001	1.01	0.02
	[95% CI]	[1.01–1.03]		[1.00–1.03]	
	**Male**	1.86	<0.001	2.16	<0.001
	[95% CI]	[1.40–2.47]		[1.57–2.96]	
**Socioeconomic and health factors**	**Completed secondary school**	0.73	<0.03	1.06	0.7
	[95% CI]	[0.55–0.96]		[0.77–1.46]	
	**BMI (kg/m^2^)**	0.97	0.2		
	[95% CI]	[0.92–1.01]			
	**Previous TB episode**	1.48	0.03	1.16	0.5
	[95% CI]	[1.04–2.10]		[0.79–1.71]	
	**Earnings at recruitment**	0.99	<0.001	NA	NA
	[95% CI]	[0.99–1.00]		NA	
	**Patient without paid employment**	1.42	<0.06	1.86	0.003
	[95% CI]	[0.99–2.05]		[1.23–2.79]	
	**Debts at recruitment** *	1.00	0.1	1.00	0.02
	[95% CI]	[0.99– 1.00]		[1.00–1.00]	
	**Household poverty score**	1.26	<0.001	1.25	<0.001
	[95% CI]	[1.17–1.35]		[1.15–1.36]	
**Current TB episode**	**Symptom duration**	1.003	0.002	1.00	0.06
	[95% CI]	[1.00–1.005]		[1.00–1.00]	
	**MDR TB**	1.92	0.003	1.61	<0.06
	[95% CI]	[1.25–2.96]		[0.98–2.64]	
	**Days too unwell to work prior to treatment**	1.01	<0.001	1.00	0.03
	[95% CI]	[1.00–1.02]		[1.00–1.01]	

Factors associated (*p*<0.15) with catastrophic costs in univariable regression were included in the multivariable regression analysis. 95% confidence intervals are shown in brackets. All patients (*n* = 876) had data available and entered the univariable and multivariable logistic regression analyses.

*Debt at recruitment was used in the final multivariable regression model rather than total debt (debt at recruitment plus debt at 24 wk of treatment) because only 461 patients had 24-wk debt data available.

NA, not applicable.

#### Catastrophic costs and adverse TB outcome

**Figure 5:**
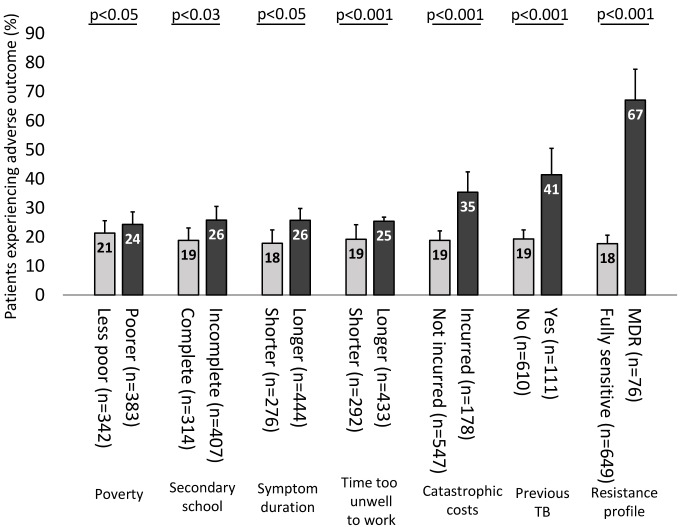
Percentage of patients experiencing an adverse TB outcome analysed by poverty, education level, symptom duration, time too unwell to work, catastrophic costs, previous TB, and resistance profile. Error bars represent 95% confidence intervals. *p*-Values correspond to the association of each variable with adverse TB outcome in univariable logistic regression, except for poverty and symptom duration, which were analysed as continuous variables. In multivariable regression analysis, the following variables remained independently associated with adverse TB outcome: time too unwell to work (*p* = 0.02), catastrophic costs (*p* = 0.003), having had a previous episode of TB (*p* = 0.004), and currently having MDR TB (*p*<0.0001).

Of the 725 patients with both TB outcome and catastrophic costs data, 166 (23%) had an adverse TB outcome. In multivariable regression analysis, having MDR TB was most strongly associated with adverse TB outcome (OR 8.4 [95% CI = 4.7–15], *p*<0.001). Having had previous TB (OR 2.1 [95% CI = 1.3–3.5], *p* = 0.005), having more days not working due to illness prior to TB diagnosis (OR 1.01 [95% CI = 1.00–1.01], *p* = 0.02), and incurring catastrophic costs (OR 1.7 [95% CI = 1.1–2.6], *p* = 0.01; Table 4 and Figure 5) were also independently associated with adverse TB outcome. When the adverse TB outcome multivariable regression analyses were repeated with total costs as a proportion of annual income analysed as a continuous outcome variable (instead of a dichotomous variable: above versus below a threshold indicating catastrophic costs), the results and patterns of significance were similar (Table S2). The likelihood ratio test did not reveal any interaction between having MDR TB and incurring catastrophic costs (whether analysed using quantiles of costs as a continuous variable or using our catastrophic costs threshold).

**Table 4:** Univariable and multivariable logistic regression of factors associated with adverse TB outcome.

Category	Characteristic	Univariable Logistic Regression		Multivariable Logistic Regression	
		OR	*p*-Value	OR	*p*-Value
**Demographics**	**Age (years)**	1.01	0.06	1.00	0.6
	[95% CI]	[1.0–1.02]		[0.99–1.02]	
	**Male**	1.53	0.02	1.25	0.3
	[95% CI]	[1.07–2.20]		[0.80–1.95]	
**Socioeconomic/health factors**	**Completed secondary school**	0.66	<0.03	0.71	0.1
	[95% CI]	[0.46–0.95]		[0.45–1.11]	
	**BMI (kg/m^2^)**	0.93	0.01	0.95	0.2
	[95% CI]	[0.87–0.98]		[0.89–1.03]	
	**Previous TB episode**	2.95	<0.001	2.11	0.005
	[95% CI]	[1.92–4.52]		[1.26–3.54]	
	**Monthly income at recruitment (Peruvian Soles)**	1.00	0.2	NA	NA
	[95% CI]	[0.99–1.00]			
	**Patient without paid employment at treatment initiation**	1.47	0.1	1.25	0.5
	[95% CI]	[0.79–1.02]		[0.70–2.24]	
	**Debt at recruitment** *	1.00	0.1	1.00	0.7
	[95% CI]	[0.99–1.00]		[0.89–1.12]	
	**Household poverty score**	1.10	<0.05	1.00	1.0
	[95% CI]	[1.00–1.20]		[1.00–1.01]	
**Current tuberculosis illness**	**MDR TB**	8.38	<0.001	8.37	<0.001
	[95% CI]	[5.04–13.93]		[4.67–15.0]	
	**Symptom duration**	1.00	<0.05	1.00	0.7
	[95% CI]	[1.00–1.01]		[1.00–1.01]	
	**Days too unwell to work prior to treatment**	1.01	<0.001	1.01	0.02
	[95% CI]	[1.00–1.01]		[1.00–1.01]	
	**Catastrophic TB costs (20% or more of annual income)**	2.36	<0.001	1.72	0.01
	[95% CI]	[1.62–3.43]		[1.11–2.64]	

Adverse TB outcome was defined as death during treatment, treatment failure or abandonment, or recurrence of TB within 30 mo of starting treatment. Factors associated (*p*<0.15) with adverse TB outcome in univariable regression were included in the multivariable regression analysis. 725/876 (83%) of patients had TB outcome data available and entered the univariable and multivariable logistic regression analyses.

*Debt at recruitment was used in the final multivariable regression model rather than total debt (the sum of debt at recruitment plus debt at 24 wk of treatment) because only 461 patients had 24-wk debt data available.

NA, not applicable.

#### Population attributable fraction

The unadjusted population attributable fraction of adverse TB outcomes explained by catastrophic costs was 26% (95% CI = 14%–36%), similar to that of MDR TB (23% [95% CI = 17%–28%]). When catastrophic costs, MDR TB, and previous TB episode were included in the multivariable regression model, the adjusted population attributable fraction of adverse TB outcomes explained by catastrophic costs and MDR TB was similar: 18% (95% CI = 6.9%–28%) for catastrophic costs and 20% (95% CI = 14%–25%) for MDR TB.

#### Sensitivity analysis of other catastrophic costs thresholds

Using a threshold of total costs of 10% or more of annual income, 578 patients (66%) incurred catastrophic costs. When this threshold was increased to total costs of 15% or more of annual income, 457 patients (52%) incurred catastrophic costs. Finally, at a threshold of total costs of 25% or more of annual income, 281 patients (32%) incurred catastrophic costs. When these thresholds were included in the multivariable regression models, it was found that catastrophic costs at a threshold of 10% or more, or 15% or more, of annual income were not independently associated with adverse TB outcome (Tables S3 and S4). Conversely, catastrophic costs at a threshold of 25% or more of annual income were independently associated with adverse TB outcome (Table S5).

## Discussion

In this prospective cohort study of TB patients in impoverished Peruvian shantytowns, accessing free TB care was expensive for poor TB patients, especially those with MDR TB. Our novel findings also define an evidence-based threshold for catastrophic costs of total costs greater than or equal to 20% of annual income for TB-affected households. Moreover, an additional sensitivity analysis revealed that other recognised catastrophic costs thresholds (such as expenses equal to or greater than 10% or 15% of annual income) did not identify any association between catastrophic costs and adverse TB outcome in this setting. Our new definition is innovative in demonstrating the strong association between this 20% catastrophic costs threshold and the increased chances of adverse TB outcome, independent of MDR TB status. Our population attributable fraction analysis supports this observation by indicating that a similar proportion of adverse TB outcomes may be averted by eliminating catastrophic costs or MDR TB from the study population. Overall, our findings suggest that the costs imposed on TB-affected households are not only financially but also clinically relevant, and highlight the importance of household poverty and catastrophic TB-related costs in relation to TB epidemiology, outcome, and control.

Social determinants are important in the causal pathway of TB disease [39]. Indeed, reducing poverty, advocating improved equity of access and universal healthcare, and eliminating catastrophic costs in TB-affected households are key components of the World Health Organization's post-2015 global TB strategy [13],[14],[26],[40]. Our results demonstrate that both drug-susceptible and MDR TB predominantly affect the poor [3],[4],[41],[42]. Our poverty score used time-stable variables such as household assets, education level, and housing [43]; thus, poverty can be assumed to have preceded TB disease. The extent and effect of poverty may be underestimated in our study given that geographical and socioeconomic barriers and stigma may particularly preclude the poorest people from seeking healthcare [44].

As in previous studies [8],[9],[11], poorer people incurred the most catastrophic costs, which they were less able to afford, probably causing further impoverishment [7],[44]. In agreement with other findings, our results indicate that costs as a proportion of the same household's income indicate economic challenge better than actual monetary expenditures: this finding highlights the “medical poverty trap”, that as incomes decrease, proportional costs increase [6],[45]. In addition to exemplifying this medical poverty trap, our results also show that being poorer was independently associated with incurring catastrophic costs. Catastrophic costs in poor households can lead to financial shock: families reducing consumption below minimum needs, selling assets, and taking children out of education. These actions may in turn increase stigmatisation [17],[18],[46],[47]. Moreover, TB principally affects the most economically productive age group, and patient and household income decreases post-diagnosis [6] and may not return to pre-diagnosis levels. Our study adds a new dimension to the social protection TB literature by showing how catastrophic costs can also have significant clinical implications: loss of income and higher hidden costs have previously been associated with poor treatment adherence and high dropout rates in TB patients [9],[44],[48], but their independent effects on long-term TB outcome have not been previously characterised. We hypothesize that the relationship we found between catastrophic costs and adverse TB outcome may relate to a number of factors along the causal pathway from TB susceptibility to illness to recurrence, including inadequate nutrition due to lower food spending, more severe disease (both MDR and non-MDR TB), and barriers to cure due to the disproportionate hidden costs associated with adherence to and completion of treatment. These adverse TB outcomes associated with catastrophic costs may increase TB and MDR-TB transmission, especially in poorer households. Thus, catastrophic costs may worsen TB control.

Regardless of the mechanisms mediating the association between catastrophic costs and adverse TB outcome, the policy implications of our findings are clear: future TB prevention strategies should incorporate social protection to mitigate decreased economic production, loss of employment, and TB-associated poverty, and to reduce the clinical vulnerability of TB patients. These findings highlight the potential role of social protection not just as a poverty-reduction strategy, but also as a tool to improve disease control and, ultimately, health [23],[24].

Our previous social protection intervention project, Innovative Socioeconomic Interventions Against Tuberculosis (ISIAT), provided evidence that in Peru multidisciplinary social protection intervention can improve adherence and completion of TB treatment and prophylaxis [35]. Social protection interventions targeting disadvantaged and vulnerable populations have shown much promise in Latin America [49], for example, conditional cash transfer projects such as the Programa de Educación, Salud y Alimentación (Progresa) in Mexico [50]. In order for these programs to be adopted on a larger scale and reduce the social health gradient, they require a rigorous evidence base that is currently lacking. Our present study suggests that by reducing catastrophic costs, social protection has the potential to protect families from TB illness and deepening poverty [17],[51].

Despite TB treatment being free of direct charges in Peru, the overall costs in TB-affected households were high and were similar pre-treatment and during treatment. Regardless of MDR TB status, higher total expenses were incurred in households where the patient had longer symptom duration prior to diagnosis, consistent with the known association between increased expenses and diagnostic delay [40]. A strength of our study is that it analysed patients with both MDR and non-MDR TB and found that expenses as a proportion of household annual income were significantly higher in patients with MDR TB, as has been noted in other Latin American countries [52],[53].

Medical expenses made up the largest proportion of pre-treatment direct expenses, probably because TB care was provided free of charge only when TB was being tested for or after TB was diagnosed. Consequently, formal medical care for the presenting illness was often expensive for the patient prior to TB being suspected and/or diagnosed [43],[54]. We found no evidence of healthcare providers requesting “informal” or “under the table” payments, costs that have been reported in other countries [43].

Extending findings from previous studies [6],[9],[11], lost income formed the majority of the economic burden of total expenses, and TB patients, especially those with MDR TB, were more likely than controls to have a lower income, to be without paid work, and to miss work due to TB-related illness. In addition, having more days too unwell to work due to TB illness was independently associated with having an adverse TB outcome. These results suggest that the socioeconomic and employment situation of TB patients is often precarious, and this may negatively impact their health, as has been found in another study [55]. Indeed, our finding that having more days too unwell to work was independently associated with both incurring catastrophic costs and having an adverse TB outcome suggests that TB illness in such patients may be more severe or advanced, and financial shock more likely.

Some definitions of catastrophic costs incorporate signs of financial shock, when a household is forced to employ coping mechanisms such as sacrificing basic needs, selling assets, selling household items, removing children from education, and incurring formal or informal debt [17],[18],[46],[47],[56],[57]. Others have defined costs as catastrophic when they exceed 10%–40% of annual household or individual income [7],[15],[56],[58] or 40% or more of a household's “capacity to pay” (the effective income for non-food spending [21],[22],[46],[59]–[61]), but this approach may be too narrow and potentially misleading to policy-makers because it overlooks lost income [23],[24]. A strength of the threshold of catastrophic costs that our results defined is that it includes not only out-of-pocket direct expenses but also lost income and that it is proven to be clinically relevant. Specifically, our definition was calculated from serial, prospective data [15],[18] of household expenses, actual household income [7],[58], and long-term TB outcome of a cohort of TB patients in impoverished Peruvian shantytowns. It has been estimated that 4% of households in Peru incur catastrophic health expenditure when aiming to meet overall health needs [62]. Rates of catastrophic health expenditure in our cohort were much higher than those of the general population. This may be due to TB-affected households being poorer or the TB treatment model in Peru having greater hidden costs for TB patients, or that we included lost income to calculate catastrophic costs, whereas only direct expenses were used in some other studies [62].

The sensitivity analysis we performed showed that the proportion of patient households incurring catastrophic costs was similar to the proportion found in other studies that used different thresholds: at a threshold of total costs of 10% or more of annual income, 65% of our cohort incurred catastrophic costs, compared to 66%–75% in related studies from sub-Saharan Africa [12],[16]; at thresholds of total costs of 15% and 25% or more of annual income, 52% and 32% of our cohort, respectively, had catastrophic costs, compared to 68% and 48%, respectively, in a cohort from sub-Saharan Africa [12]. More importantly, the sensitivity analysis also showed that thresholds of total costs of 10% and 15% or more of annual income were not independently associated with adverse TB outcome in this Peruvian shantytown setting. Our results demonstrate that these previously published arbitrary thresholds for catastrophic costs that were defined without patient follow-up were not associated with adverse TB outcome for TB patients in our setting. Thus, our findings provide, to our knowledge, the first evidence-based threshold for clinically relevant catastrophic costs, and demonstrate a methodology to assess the generalizability of this threshold in other settings.

This study has several limitations. First, cases and controls were not matched in this study because controls were specifically included to provide an estimate of typical income and expenditure in this community, to be compared with TB patients at baseline. Matching would have impaired this comparison. A baseline difference was noted in debts at recruitment, a proxy for “dis-saving”. Poorer households have diminished access to establishments that offer formal loans (e.g., banks) because of their uncertain repayment capacity and/or lack of requisites such as a national identity card [35]. However, even if controls had been matched to cases, controls may still have had higher debt than patients because some patient households were not eligible for some loans due to serious ill health or if they were extremely poor [35]. Apart from debt, no other data were collected on specific “dis-saving” coping mechanisms. However, although selling household items may have been overlooked, taking children out of education and selling livestock are unlikely to occur in the peri-urban non-agrarian communities that made up our study cohort. Second, the data available did not allow assessment of an existing WHO definition of catastrophic costs (40% or more of a household's capacity to pay [63]) against which other studies have compared their findings [12]. Third, we may have underestimated the financial effects of MDR TB because our questionnaires quantifying costs continued for only 6 mo, whereas patients with MDR TB are usually treated for 18 mo or more. We decided a priori to analyse the catastrophic costs of both MDR and non-MDR patients together, given their equal follow-up and the small number of MDR TB patients. Finally, our research demonstrates a new methodology that should be repeated in other settings to assess the external validity of our findings.

Despite free TB care, having TB disease was expensive for TB patients living in a shantytown in Peru. Higher relative costs were associated with greater likelihood of adverse TB outcome. Having MDR TB and incurring catastrophic costs were independently associated with adverse TB outcome, with a similar adjusted population attributable fraction for adverse TB outcome. Thus, catastrophic costs were an indicator of both financial and clinical vulnerability, and households affected by TB would benefit from assessment to identify those at highest risk of incurring catastrophic costs. Mitigating catastrophic costs through targeted social protection interventions as well as prompt diagnosis and appropriate treatment of MDR TB deserve attention in TB control programs. In conclusion, control interventions must consider TB as an infectious and socioeconomic problem and address both the clinical and financial aspects of this public health challenge.

## Supporting Information

Figure S1 **Lost income, direct expenses, and total expenses by treatment stage in mean Peruvian Soles and as a proportion of mean monthly household income.** A line chart representation of the data presented in Figure 1.
(TIF)

Table S1 **Factors associated with total costs as a proportion of annual income.** Total costs as a proportion of annual income had a non-Gaussian distribution, so this variable was transformed to its base-10 logarithm for regression analysis. Factors associated (*p*<0.15) with increasing costs in univariable linear regression were included in the multivariable analysis. 95% confidence intervals are shown in parentheses. All patients (*n* = 876) had data available and were included in the univariable and multivariable linear regression analyses.
(DOC)

Table S2 **Univariable and multivariable logistic regression of factors (including costs as a continuous variable) associated with adverse outcome.** Adverse outcome is defined as death during treatment, treatment failure or abandonment, or recurrence of TB within 30 mo of starting treatment. Total costs as a proportion of annual income had a non-Gaussian distribution so this variable was transformed to its base-10 logarithm for regression analysis. Factors associated (*p*<0.15) with adverse outcome in univariable logistic regression were included in the multivariable logistic regression analysis. 725/876 (83%) of patients had outcome data available and were included in the univariable and multivariable logistic regression analyses. This table differs from Table 4 in that total costs as a proportion of annual income is analysed as a continuous variable instead of as a dichotomous variable (catastrophic versus non-catastrophic costs).
(DOC)

Table S3 **Univariable and multivariable logistic regression of factors (including 10% threshold for catastrophic costs) associated with adverse outcome.** Adverse outcome is defined as death during treatment, treatment failure or abandonment, or recurrence of TB within 30 mo of starting treatment. Factors associated (*p*<0.15) with adverse outcome in univariable logistic regression were included in the multivariable logistic regression analysis. 725/876 (83%) of patients had outcome data available and entered the univariable and multivariable logistic regression analyses. In contrast to Table 4, in this table total costs ≥10% of annual income was used as the threshold for catastrophic costs.
(DOC)

Table S4 **Univariable and multivariable logistic regression of factors (including 15% threshold for catastrophic costs) associated with adverse outcome.** Adverse outcome is defined as death during treatment, treatment failure or abandonment, or recurrence of TB within 30 mo of starting treatment. Factors associated (*p*<0.15) with adverse outcome in univariable logistic regression were included in the multivariable logistic regression analysis. 725/876 (83%) of patients had outcome data available and entered the univariable and multivariable logistic regression analyses. In contrast to Table 4, in this table total costs ≥15% of annual income was used as the threshold for catastrophic costs.
(DOC)

Table S5 **Univariable and multivariable logistic regression of factors (including 25% threshold for catastrophic costs) associated with adverse outcome.** Adverse outcome is defined as death during treatment, treatment failure or abandonment, or recurrence of TB within 30 mo of starting treatment. Factors associated (*p*<0.15) with adverse outcome in univariable logistic regression were included in the multivariable logistic regression analysis. 725/876 (83%) of patients had outcome data available and were included in the univariable and multivariable logistic regression analyses. In contrast to Table 4, in this table total costs ≥25% of annual income was used as the threshold for catastrophic costs.
(DOC)

Table S6 **Annual inflation rate of the Peruvian Sol and exchange rate of the Peruvian Sol.** Source: l PEN to the US dollar, 2002–2009 [64].
(DOC)

Questionnaire S1 **Socioeconomic section of initial and follow-up questionnaires.**
(DOC)

## Abbreviations

BMI: body mass index
MDR: multi-drug-resistant
OR: odds ratio
PEN: Peruvian Soles
TB: tuberculosis
