# Supplementary figures and images for: Defining Catastrophic Costs and Comparing Their Importance for Adverse Tuberculosis Outcome with Multi-Drug Resistance: A Prospective Cohort Study, Peru
Source: PLoS Med. 2014 Jul 15;11(7):e1001675. doi: 10.1371/journal.pmed.1001675 (PMC4098993; doi:10.1371/journal.pmed.1001675)

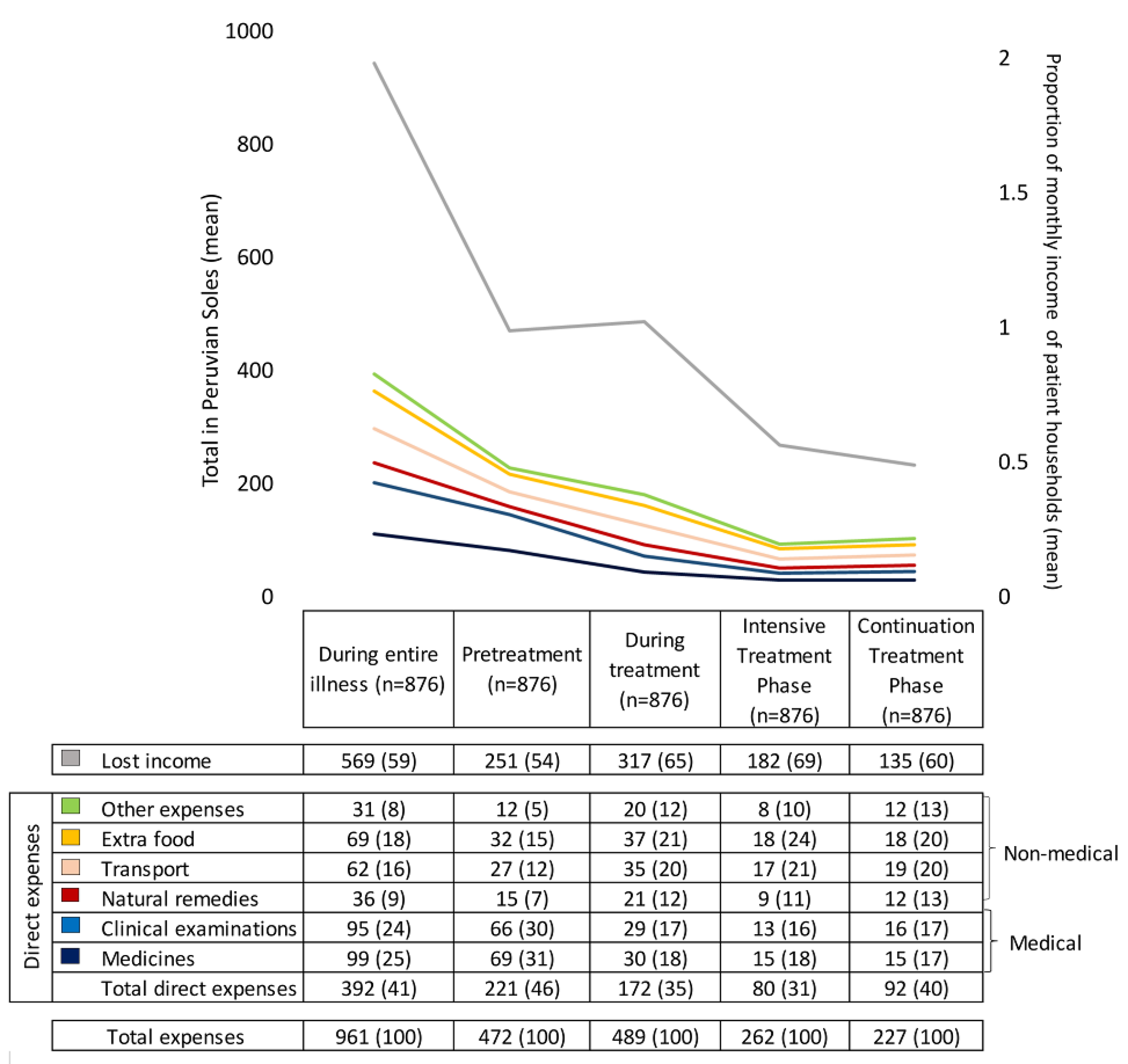

Supplement: Figure S1 — Lost income, direct expenses, and total expenses by treatment stage in mean Peruvian Soles and as a proportion of mean monthly household income. A line chart representation of the data presented in Figure 1. (TIF) [file pmed.1001675.s001.tif]
