# Supplementary material for: Defining Catastrophic Costs and Comparing Their Importance for Adverse Tuberculosis Outcome with Multi-Drug Resistance: A Prospective Cohort Study, Peru
Source: PLoS Med. 2014 Jul 15;11(7):e1001675. doi: 10.1371/journal.pmed.1001675 (PMC4098993; doi:10.1371/journal.pmed.1001675)
Supplement: Table S1 — Factors associated with total costs as a proportion of annual income. Total costs as a proportion of annual income had a non-Gaussian distribution, so this variable was transformed to its base-10 logarithm for regression analysis. Factors associated (p<0.15) with increasing costs in univariable linear regression were included in the multivariable analysis. 95% confidence intervals are shown in parentheses. All patients (n = 876) had data available and were included in the univariable and multivariable linear regression analyses. (DOC) [file pmed.1001675.s002.doc]

**Supplementary Table 1: Factors associated with total costs as a proportion of annual income.** Total costs as a proportion of annual income data had a non-Gaussian distribution so this variable was transformed to its base-10 logarithm for regression analysis. Factors associated (p<0.15) with increasing costs in univariable linear regression were included in the multivariable analysis. 95% confidence intervals are shown in parentheses. All patients (n=876) had data available and were included in the univariable and multivariable linear regression analyses.

|  |  |  |  |  |  |
| --- | --- | --- | --- | --- | --- |
|  | Univariable | |  | Multivariable logistic regression | |
|  | Coefficient | p |  | Coefficient | p |
| *Demographics* |  |  |  |  |  |
| **Age; mean years [95% CI]** | 0.0053 | <0.001 |  | 0.0038 | 0.002 |
|  | [0.0028-0.077] |  |  | [0.0014-0.0061] |  |
| **Sex; males [95% CI]** | 0.12 | <0.001 |  | 0.12 | <0.001 |
|  | [0.056-0.19] |  |  | [0.060-0.19] |  |
|  |  |  |  |  |  |
| *Socioeconomic and health factors* |  |  |  |  |  |
| **Completed secondary school; [95% CI]** | -0.19 | <0.001 |  | -0.012 | 0.7 |
|  | [-0.18 - -0.054] |  |  | [-0.078-0.055] |  |
| **BMI; [95% CI]** | -0.0056 | 0.3 |  | . | . |
|  | [-0.016 - 0.047] |  |  | . |  |
| **Previous TB episode; [95% CI]** | 0.12 | 0.004 |  | 0.074 | 0.08 |
|  | [0.039-0.21] |  |  | [-0.0077-0.16] |  |
| **Earnings at recruitment; [95% CI]** | -0.00029 | <0.001 |  | NA | NA |
|  | [-0.00036 - -0.00021] |  |  | NA |  |
| **Patient without paid employment; [95% CI]** | 0.094 | <0.03 |  | 0.13 | 0.001 |
|  | [0.010-0.18] |  |  | [0.031-0.065] |  |
| **Debts at recruitment; [95% CI]** | 0.000014 | 0.2 |  | . | . |
|  | [0.000006 – 0.000038] |  |  | . |  |
| **Poverty; [95% CI] household poverty score** | 0.060 | <0.001 |  | 0.048 | <0.001 |
|  | [0.044-0.076] |  |  | [0.034-0.067] |  |
| *Current tuberculosis episode* |  |  |  |  |  |
| **Symptom duration; % [95% CI]** | 0.0011 | <0.001 |  | 0.00080 | <0.001 |
|  | [0.00070-0.0016] |  |  | [0.00037-0.0012] |  |
| **MDR; % [95% CI]** | 0.17 | 0.001 |  | 0.075 | 0.2 |
|  | [0.068-0.29] |  |  | [-0.028-0.18] |  |
| **Days too unwell to work prior to treatment; [95% CI]** | 0.0024 | <0.001 |  | 0.0010 | <0.05 |
|  | [0.0014-0.0034] |  |  | [0.000059-0.0020] | . |
|  |  |  |  |  |  |
|  |  |  |  |  |  |
