# Supplementary material for: Defining Catastrophic Costs and Comparing Their Importance for Adverse Tuberculosis Outcome with Multi-Drug Resistance: A Prospective Cohort Study, Peru
Source: PLoS Med. 2014 Jul 15;11(7):e1001675. doi: 10.1371/journal.pmed.1001675 (PMC4098993; doi:10.1371/journal.pmed.1001675)
Supplement: Table S2 — Univariable and multivariable logistic regression of factors (including costs as a continuous variable) associated with adverse outcome. Adverse outcome is defined as death during treatment, treatment failure or abandonment, or recurrence of TB within 30 mo of starting treatment. Total costs as a proportion of annual income had a non-Gaussian distribution so this variable was transformed to its base-10 logarithm for regression analysis. Factors associated (p<0.15) with adverse outcome in univariable logistic regression were included in the multivariable logistic regression analysis. 725/876 (83%) of patients had outcome data available and were included in the univariable and multivariable logistic regression analyses. This table differs from Table 4 in that total costs as a proportion of annual income is analysed as a continuous variable instead of as a dichotomous variable (catastrophic versus non-catastrophic costs). (DOC) [file pmed.1001675.s003.doc]

**Supplementary Table 2: Univariable and multivariable logistic regression of factors (including costs as a continuous variable) associated with adverse outcome.** Adverse outcome is defined as death during treatment, treatment failure or abandonment, and recurrence of TB within 30 months of starting treatment. Total costs as a proportion of annual income data had a non-Gaussian distribution so this variable was transformed to its base-10 logarithm for regression analysis. Factors associated (p<0.15) with adverse outcome in univariable logistic regression were included in the multivariable logistic regression analysis. 725/876 (83%) of patients had outcome data available and were included in the univariable and multivariable logistic regression analyses. This supplementary table differs from Table 4 of the main manuscript because total costs as a proportion of annual income is analysed as a continuous variable instead of as a dichotomous variable (catastrophic costs).

|  |  |  |  |  |  |
| --- | --- | --- | --- | --- | --- |
|  | Univariable | |  | Multivariable logistic regression | |
|  | OR | p |  | OR | p |
|  |  |  |  |  |  |
| *Demographics* |  |  |  |  |  |
| **Age; mean years [95% CI]** | 1.01 | 0.06 |  | 1.00 | 0.7 |
|  | [1.0-1.02] |  |  | [0.99-1.02] |  |
| **Sex; males [95% CI]** | 1.53 | 0.02 |  | 1.26 | 0.3 |
|  | [1.07-2.20] |  |  | [0.81-1.95] |  |
|  |  |  |  |  |  |
| *Socioeconomic and health factors* |  |  |  |  |  |
| **Completed secondary school; [95% CI]** | 0.66 | <0.03 |  | 0.72 | 0.2 |
|  | [0.46-0.95] |  |  | [0.46-1.13] |  |
| **BMI; [95% CI]** | 0.93 | 0.01 |  | 0.95 | 0.2 |
|  | [0.96-1.00] |  |  | [0.89-1.02] |  |
| **Previous TB episode; [95% CI]** | 2.95 | <0.001 |  | 2.07 | 0.006 |
|  | [1.92-4.52] |  |  | [1.24-3.48] |  |
| **Income at recruitment; per month in Peruvian soles; [95% CI]** | 1.00 | 0.2 |  | NA | NA |
|  | [0.99 - 1.00] |  |  |  |  |
| **Patient without paid employment at treatment; [95% CI]** | 1.47 | 0.1 |  | 1.24 | 0.5 |
|  | [0.79-1.02] |  |  | [0.69-2.22] |  |
| **Debts; [95% CI]** | 1.00 | 0.1 |  | 1.00 | 0.8 |
|  | [0.99-1.00] |  |  | [1.00-1.00] |  |
| **Poverty; [95% CI] household poverty score** | 1.10 | <0.05 |  | 1.00 | 1.0 |
|  | [1.00-1.20] |  |  | [1.00-1.00] |  |
| *Current tuberculosis illness* |  |  |  |  |  |
| **MDR; [95% CI]** | 8.38 | <0.001 |  | 8.38 | <0.001 |
|  | [5.04-13.93] |  |  | [4.68-15.0] |  |
| **Symptom duration; [95% CI]** | 1.00 | <0.05 |  | 1.0 | 0.7 |
|  | [1.00-1.01] |  |  | [1.00-1.00] |  |
| **Days too unwell to work prior to treatment; [95% CI]** | 1.01 | <0.001 |  | 1.00 | 0.2 |
|  | [1.00-1.01] |  |  | [1.00-1.01] |  |
| **Total costs as a proportion of annual income (log transformation)*; [95% CI]** | 2.53 | <0.001 |  | 1.84 | 0.007 |
|  | [1.77-3.61] |  |  | [1.18-2.88] |  |
|  |  |  |  |  |  |

*if the non-Gaussian original total costs as a proportion of annual income is used instead of the transformation to base-10 logarithm the association is similar: Univariable logistic regression OR 1.69 [95% CI 1.33-2.15] p<0.001, multivariable logistic regression (same pattern of significance as shown) OR 1.56 [95% CI 1.22-1.98], p<0.001
