# Supplementary material for: Defining Catastrophic Costs and Comparing Their Importance for Adverse Tuberculosis Outcome with Multi-Drug Resistance: A Prospective Cohort Study, Peru
Source: PLoS Med. 2014 Jul 15;11(7):e1001675. doi: 10.1371/journal.pmed.1001675 (PMC4098993; doi:10.1371/journal.pmed.1001675)
Supplement: Table S5 — Univariable and multivariable logistic regression of factors (including 25% threshold for catastrophic costs) associated with adverse outcome. Adverse outcome is defined as death during treatment, treatment failure or abandonment, or recurrence of TB within 30 mo of starting treatment. Factors associated (p<0.15) with adverse outcome in univariable logistic regression were included in the multivariable logistic regression analysis. 725/876 (83%) of patients had outcome data available and were included in the univariable and multivariable logistic regression analyses. In contrast to Table 4, in this table total costs ≥25% of annual income was used as the threshold for catastrophic costs. (DOC) [file pmed.1001675.s006.doc]

**Supplementary Table 5: Univariable and multivariable logistic regression of factors (including 25% threshold catastrophic costs) associated with adverse outcome.** Adverse outcome is defined as death during treatment, treatment failure or abandonment, and recurrence of TB within 30 months of starting treatment. Factors associated (p<0.15) with adverse outcome in univariable logistic regression were included in the multivariable logistic regression analysis. 725/876 (83%) of patients had outcome data available and were included in the Univariable and Multivariable logistic regression analyses. In contrast to Table 4 of the main manuscript, in this table total costs ≥25% of annual income were used as the threshold for catastrophic costs

|  |  |  |  |  |  |
| --- | --- | --- | --- | --- | --- |
|  | Univariable | |  | Multivariable logistic regression | |
|  | OR | p |  | OR | P |
|  |  |  |  |  |  |
| *Demographics* |  |  |  |  |  |
| **Age; mean years [95% CI]** | 1.01 | 0.06 |  | 1.00 | 0.8 |
|  | [1.0-1.02] |  |  | [0.99-1.02] |  |
| **Sex; males [95% CI]** | 1.53 | 0.02 |  | 1.23 | 0.4 |
|  | [1.07-2.20] |  |  | [0.79-1.92] |  |
|  |  |  |  |  |  |
| *Socioeconomic and health factors* |  |  |  |  |  |
| **Completed secondary school; [95% CI]** | 0.66 | <0.03 |  | 0.70 | 0.1 |
|  | [0.46-0.95] |  |  | [0.45-1.10] |  |
| **BMI; [95% CI]** | 0.93 | 0.01 |  | 0.95 | 0.2 |
|  | [0.96-1.00] |  |  | [0.89-1.03] |  |
| **Previous TB episode; [95% CI]** | 2.95 | <0.001 |  | 2.18 | 0.003 |
|  | [1.92-4.52] |  |  | [1.30-3.65] |  |
| **Income at recruitment; per month in Peruvian soles; [95% CI]** | 1.00 | 0.2 |  | NA | NA |
|  | [0.99 - 1.00] |  |  |  |  |
| **Patient without paid employment at treatment; [95% CI]** | 1.47 | 0.1 |  | 1.23 | 0.5 |
|  | [0.79-1.02] |  |  | [0.69-2.20] |  |
| **Debts; [95% CI]** | 1.00 | 0.1 |  | 1.00 | 0.8 |
|  | [0.99-1.00] |  |  | [1.00-1.00] |  |
| **Poverty; [95% CI] household poverty score** | 1.10 | <0.05 |  | 1.00 | 0.9 |
|  | [1.00-1.20] |  |  | [0.89-1.13] |  |
| *Current tuberculosis illness* |  |  |  |  |  |
| **MDR; [95% CI]** | 8.38 | <0.001 |  | 8.30 | <0.001 |
|  | [5.04-13.93] |  |  | [4.63-14.9] |  |
| **Symptom duration; [95% CI]** | 1.00 | <0.05 |  | 1.00 | 0.8 |
|  | [1.00-1.01] |  |  | [1.00-1.00] |  |
| **Days too unwell to work prior to treatment; [95% CI]** | 1.01 | <0.001 |  | 1.00 | 0.2 |
|  | [1.00-1.01] |  |  | [1.00-1.01] |  |
| **Catastrophic costs threshold ≥25% of annual income; [95% CI]** | 2.59 | <0.001 |  | 1.98 | 0.002 |
|  | [1.81-3.71] |  |  | [1.28-3.07] |  |
|  |  |  |  |  |  |
|  |  |  |  |  |  |
