# Supplementary material for: Defining Catastrophic Costs and Comparing Their Importance for Adverse Tuberculosis Outcome with Multi-Drug Resistance: A Prospective Cohort Study, Peru
Source: PLoS Med. 2014 Jul 15;11(7):e1001675. doi: 10.1371/journal.pmed.1001675 (PMC4098993; doi:10.1371/journal.pmed.1001675)
Supplement: Table S6 — Annual inflation rate of the Peruvian Sol and exchange rate of the Peruvian Sol. Source: l PEN to the US dollar, 2002–2009 [64]. (DOC) [file pmed.1001675.s007.doc]

**Supplementary Table 6: Annual inflation rate of the Peruvian Sol and exchange rate of the Peruvian Sol to the United States (US)** Dollar, 2002-2009

| Year | Inflation rate (%) | Exchange rate Peruvian Soles to US Dollar |
| --- | --- | --- |
| 2002 | 0.192 | 0.284 |
| 2003 | 2.261 | 0.287 |
| 2004 | 3.662 | 0.432 |
| 2005 | 1.618 | 0.484 |
| 2006 | 2.001 | 0.305 |
| 2007 | 1.779 | 0.319 |
| 2008 | 5.788 | 0.342 |
| 2009 | 2.935 | 0.334 |

Source: International Monetary Fund –World economic outlook 2011: a survey by the staff of the International Monetary Fund. Washington, DC: International Monetary Fund, 1980-2011; ISBN 978-1-61635-119-9

<http://www.imf.org/external/pubs/ft/weo/2011/02/pdf/text.pdf>
