# Supplementary material for: Defining Catastrophic Costs and Comparing Their Importance for Adverse Tuberculosis Outcome with Multi-Drug Resistance: A Prospective Cohort Study, Peru
Source: PLoS Med. 2014 Jul 15;11(7):e1001675. doi: 10.1371/journal.pmed.1001675 (PMC4098993; doi:10.1371/journal.pmed.1001675)
Supplement: Questionnaire S1 — Socioeconomic section of initial and follow-up questionnaires. (DOC) [file pmed.1001675.s008.doc]

**Supplementary Date File. Socio-economic section of initial and follow up questionnaires**

75) ¿Cuántos días estuvo muy enfermo como para continuar con sus actividades cotidianas antes de empezar el tratamiento? *(How long were you too sick to continue working/studying before the beginning of therapy?)*: _____ días

1. ¿Cuán endeudada está su familia? *(How much debt does your family have?)*: _____ S/.
2. Su vivienda es... *(Tenancy)*:

a. Propia

b. Alquilada

c. Prestada

d. Hipotecada

e. Otro: ________________________________

1. ¿Hasta qué grado estudió el jefe/la jefa de su familia? *(What’s the level of education of the head of the household?)*:

a. Analfabeto

b. Primaria

c. Secundaria o técnica incompleta

d. Secundaria o técnica completa

e. Superior incompleta

f. Superior completa

1. ¿Cuántas personas normalmente duermen en casa? *(How many people normally sleep at home?)*: _____ personas
2. ¿Qué material predomina en las paredes exteriores de su vivienda? *(Which material predominates on the exterior walls of your house?)*:

a. Ladrillo, cemento

b. Adobe

c. Quincha (caña con barro)

d. Piedra con barro

e. Madera

f. Estera

g. Otro: _______________________________

1. ¿Qué material predomina en los pisos de su vivienda? *(Which material predominates on the floors of your house?)*:

a. Parquet, madera pulida

b. Láminas asfálticas, viníilicos, similar

c. Losetas, terrazos, similares

d. Madera, entablado

e. Cemento

f. Tierra, arena, ripio

g. Otro: _______________________________

1. ¿Cuántas habitaciones hay en su hogar? (sin contar baño, pasadizo, cocina, depósito, garage) *(How many rooms in your house?—without considering the bathroom, hallways, kitchen, storage, garage)*: ____ habitaciones
2. ¿Cómo se abastece de agua su hogar? *(How is water supplied to your house?)*:

a. Red pública dentro de vivienda

b. Red pública fuera de vivienda

c. Pilón

d. Pozo

e. Río, acequia, puquio

f. Camión tanque, aguatero

g. Agua entubada no potable

h. Otro: _______________________________

1. ¿Qué tipo de servicio higiénico tiene? *(What kind of bathroom do you have* *at home?)*:

a. A red pública dentro de vivienda

b. A red pública fuera de vivienda

c. Letrina

d. Pozo ciego séptico, pozo ciego negro, silo

e. Sobre acequia, canal

f. No tiene servicio higiénico

g. Otro: _______________________________

1. ¿Comparte el servicio higiénico con miembros de otra vivienda? *(Do you share your bathroom with members of another household?)*:

Sí No

1. ¿Qué tipo de alumbrado tiene su hogar? *(What kind of lighting do you have at home?)*:

a. Electricidad

b. Kerosene, petróleo, gas

c. Vela

d. Ninguno

e. Otro: ________________________________

1. ¿Qué combustible usan para cocinar? *(Which type of fuel do you use for cooking?)*:

a. Electricidad

b. Kerosene, petróleo, gas

c. Carbón

d. Leña

e. No cocina

f. Otro: ________________________________

Tienen en casa... *(Do you have at home...)*

1. TV que funcione: Sí No
2. Radio que funcione: Sí No
3. Teléfono que funcione: Sí No
4. Cocina que funcione: Sí No
5. Refrigerador que funcione: Sí No

¿Cuánto dinero le ha costado a usted y a su familia el que usted tenga TBC, desde que le comenzaron los síntomas? *(How much has having TB cost you and your family since the symptoms began?):*

1. Remedios naturales: ____ S/.
2. Comprar más comida: ____ S/.
3. Pérdida de ingresos en la familia: ____ S/.
4. Movilidad (taxis, micros, etc.): ____ S/.
5. Exámenes clínicos (rayos X, muestras clínicas, consultas, etc): ____ S/.
6. Medicinas: ____ S/.
7. Otros gastos: ____ S/.

(CONTINUED ON NEXT PAGE)

1. ¿Qué actividad principal se encontraba realizando la semana pasada? *(What was the main* *activity you were performing last week?)*:

a. Trabajo dependiente, regularmente remunerado (sueldo)

b. No trabajó pero tenía trabajo regularmente remunerado (sueldo)

c. Muy enfermo como para realizar tareas usuales

d. Trabajo familiar no remunerado

e. Trabajo independiente (ambulante, taxista, en mercado, etc)

k. Buscando trabajo

m. Labores domésticas / en casa

n. Estudiando, en vacaciones del centro educat.

q. Otro: _______________________________

1. ¿Cuál es el ingreso mensual de la vivienda? *(What is the monthly household income?)*: _____ S/.
2. ¿Cuánto gasta su familia en alimentación cada semana? *(How much does your family spend in food every week?)*: _____ S/.

¿Cuántas personas en su vivienda comen de esos alimentos que compran semanalmente? *(How many people in your household eat from the food that is bought every week?)*: _____ personas

**Seguimiento: 2da Semana**

1. Número de días desde la última entrevista, *(# of days since last interview)*: _____ días

¿Cuánto dinero le ha costado a usted y a su familia el que usted tenga TBC, desde la última entrevista? *(How much has having TB cost you and your family since the last interview?):*

1. Remedios naturales: ____ S/.
2. Comprar más comida: ____ S/.
3. Pérdida de ingresos en la familia: ____ S/.
4. Movilidad (taxis, micros, etc.): ____ S/.
5. Exámenes clínicos (rayos X, muestras clínicas, consultas, etc): ____ S/.
6. Medicinas: ____ S/.
7. Otros gastos: ____ S/.
8. ¿Qué actividad principal se encontraba realizando la semana pasada? *(What was the main* *activity you were performing last week?)*:

a. Trabajo dependiente, regularmente remunerado (sueldo)

b. No trabajó pero tenía trabajo regularmente remunerado (sueldo)

c. Muy enfermo como para realizar tareas usuales

d. Trabajo familiar no remunerado

e. Trabajo independiente (ambulante, taxista, en mercado, etc)

k. Buscando trabajo

m. Labores domésticas / en casa

n. Estudiando, en vacaciones del centro educat.

q. Otro: _______________________________

1. ¿Cuál es el ingreso mensual de la vivienda? *(What is the monthly household income?)*: _____ S/.
2. ¿Cuánto gasta su familia en alimentación cada semana? *(How much does your family spend in food every week?)*: _____ S/.
3. ¿Cuántas personas en su vivienda comen de esos alimentos que compran semanalmente? *(How many people in your household eat from the food that is bought every week?)*: _____ personas
